# Reviewed article: Baggio AC, Mahana G, Oliveira C, Campos JC, Trevisol DJ, Schuelter-Trevisol F, Traebert E, Traebert J. Self-perception of depressive symptoms, self-reported medical diagnosis, and associated factors: findings from the Brazilian Longitudinal Study of Aging, 2019-2021. Epidemiol Serv Saude. 2026:35;e20250158

**DOI:** 10.1590/S2237-96222026v35e20250158.b

**Published:** 2026-01-16

**Authors:** Marilia Mastrocolla de Almeida Cardoso

**Affiliations:** 1 Universidade do Sul de Santa Catarina, Tubarão, SC, Brazil

## First round comments

Prezados autores, e complemento ao que foi apontado pelos revisores, seguem abaixo alguns comentários.

Tanto no resumo, como no texto, todas as medidas de prevalência devem ser acompanhadas de IC 95%.

Remover do resumo e do texto todos os textos intepretativos “2,23 vezes mais prevalência” - Evite texto pouco específico como “foi associado” ou “encontrou-se associação”, informar a direção da associação por meio de texto informativo, como “o desfecho foi maior em crianças”, apresentando imediatamente a medida de associação e intervalo de confiança, sem interpretações como “foi duas vezes maior.

Inserir tipo de estudo na palavras-chave. Sugestão, substituir “depressão” ou “sintomas depressivos” por “Estudos Transversais”

Aderir ao guia de redação principalmente para o método e reordenar as ideias da introdução e discussão. A discussão está muito extensa e negligência limitações importantes como ausência de instrumento validado para aferir desfecho principal. Ao invés de repetir as perguntas, que é um item de métodos, deveria discutir as implicações de somente coletar dados de autorrelato.

Aspectos de redação que constam nas instruções foram negligenciados como nas seções de revisão de literatura (introdução e discussão), o foco deve ser os dados científicos. Evitar destacar organismos, autores ou nomes de relatórios, cujas informações encontram-se nas referências. Construções como “outros autores”, “outros estudos”, “a literatura aponta” etc. devem ser evitadas: apresentar o dado com clareza e citar a referência próximo à afirmação. Afirmações categóricas sobre ausência de estudos prévios devem ser evitadas em delineamentos que não sejam revisões sistemáticas da literatura. Esse aspecto não foi observado por exemplo no primeiro parágrafo da introdução. A partir deste exemplo pontual, revisar todo o texto para assegurar que adere a esse aspecto e demais itens das instruções da revista.

Siga o formato do periódico nas referências. Remover referências, pois, de acordo com as novas normas da revista, serão permitidas 30 referências no máximo.

Disponibilidade dos dados: declaração sobre o acesso aos dados de pesquisa (bancos de dados gerados para análise, códigos, métodos e outros materiais utilizados e resultantes da pesquisa), informar link do repositório e referenciamento, com a devida citação no texto; não deve ser colocado o link para o banco de dados original, mais sim aquele criado pelos autores especificamente para o estudo que deve esar disponível para acesso. Os dados de pesquisa (como bancos de dados, códigos, métodos e outros materiais utilizados ou resultantes da pesquisa) devem, preferencialmente, ser depositados em repositórios de dados confiáveis, como o SciELO Data (lista disponível em https://wp.scielo.org/wp-content/uploads/Lista-de-Repositorios-Recomendados_pt.pdf), com um identificador persistente, como o DOI (Digital Object Identifier).

O acesso a esses dados, por meio de um link para o repositório, deve ser informado na seção “Disponibilidade de dados” e a referência completa deve ser incluída na seção de “Referências”, conforme exemplo abaixo:

Andrade M. Estudo de genes em ratos albinos na América Latina. OSF [dataset], 2018, ASM0000v1. http://dx.doi.org/10.1590/0123-45620187214” 

Recommendation: Major revision

## Second round comments

Prezados autores, o texto ainda apresenta necessidade de ajustes e revisão maior para poder seguir no processo de revisão.

- Alguns trechos novos inseridos necessitam de referências para fortalecer a relevância do estudo:

“No entanto, essa estratégia apresenta limitações importantes, como a possibilidade de viés relacionado ao acesso desigual aos serviços de saúde, à compreensão dos sintomas e ao reconhecimento prévio de um diagnóstico por um profissional de saúde. Tais fatores podem influenciar tanto a subnotificação quanto a superestimação dos casos, devendo ser considerados na interpretação dos resultados.”

“Apesar da crescente atenção à saúde mental da população idosa, ainda há lacunas importantes na literatura nacional quanto à compreensão integrada do autorrelato de diagnóstico médico de depressão e da autopercepção de sintomas depressivos em idosos brasileiros. Poucos trabalhos exploraram de forma específica os sintomas depressivos na população idosa, especialmente em associação a fatores comportamentais e sociodemográficos. Além disso, ainda é limitado o número de estudos que discutem as possíveis discrepâncias entre o diagnóstico médico autorreferido e a presença de sintomas subjetivos, o que pode revelar desigualdades no acesso a diagnóstico e tratamento.”

Resultados: destacar nos resultados as principais informações que respondem aos objetivos e não somente mencionar as tabelas.

Discussão: rever cuidadosamente. O primeiro parágrafo da discussão deve resumir os achados, interpretando-os e sem repetir números nem citar ilustrações ou referências: não informar o que fez, interpretar o que encontrou.

Este trecho da conclusão resume os achados, porém apresenta dados numéricos. “Pode-se concluir que a prevalência relato de diagnóstico médico de depressão foi de 12,2% em idosos brasileiros. Do total investigado, 15,6% relataram sentir-se deprimidos, expondo uma lacuna entre relato de diagnóstico médico de depressão e o de sintomas depressivos cotidianos. Fatores como sexo feminino, sedentarismo e escolaridade intermediária mostraram-se associados a maior prevalência.”

- Alinhar objetivos, resultados com a discussão.

- O último parágrafo da conclusão (curto e conciso) deve ser revisto e apresentar a conclusão da pesquisa com base no que se pode afirmar a partir dos resultados.

Recommendation: Major revision

